# Boundary condition and oceanic impacts on the atmospheric water balance in limited area climate model ensembles

**DOI:** 10.1038/s41598-021-85744-y

**Published:** 2021-03-18

**Authors:** Klaus Goergen, Stefan Kollet

**Affiliations:** 1 Institute of Bio- and Geosciences (IBG-3, Agrosphere), Forschungszentrum Jülich, 52425 Jülich, Germany; 2 Centre for High-Performance Scientific Computing in Terrestrial Systems, Geoverbund ABC/J, 52425 Jülich, Germany

**Keywords:** Climate and Earth system modelling, Hydrology

## Abstract

Regional climate models (RCMs) are indispensable in climate research, albeit often characterized by biased terrestrial precipitation and water budgets. This study identifies excess oceanic evaporation, in conjunction with the RCMs’ boundary conditions, as drivers contributing to these biases in RCMs with forced sea surface temperatures in a CORDEX RCM ensemble over Europe. The RCMs are relaxed to the prescribed lateral boundary conditions originating from a global model, effectively matching the driving model's overall atmospheric moisture flux divergence. As a consequence, excess oceanic evaporation results in positive precipitation biases over land due to forced internal recycling of moisture to maintain the overall flux divergence prescribed by the boundary conditions. This systematic behaviour is shown through an analysis of long-term atmospheric water budgets and atmospheric moisture exchange between oceanic and continental areas in a multi-model ensemble.

## Introduction

Since the first regional climate model (RCM) simulations by Refs.^1–3^, RCMs have undergone many advancements towards Earth system modelling in climate research, as summarized by Refs.^4,5^. In dynamical downscaling setups, RCMs with their higher spatial resolution can represent small-scale surface heterogeneities due to orography, coastlines, lakes, or land cover as well as mesoscale dynamical processes or extreme events in more detail than global climate models (GCMs)^6^.

In addition, RCMs are used to answer a variety of research questions^4^, and generate climate change projections, e.g., as part of large multi model ensembles experiments, that form the basis for vulnerability, impact and adaptation studies^7,8^; the latest of which is the ongoing COordinated Regional Downscaling EXperiment (CORDEX)^9^. Over the years, many water cycle-related questions have been addressed with RCMs. For example, RCMs have been used as drivers for hydrological models assessing projected future evolution of river discharge^10–12^, to assess water budgets in catchment hydrology^13,14^, as input to groundwater models^15–17^, in hydropower modelling^18–20^, or to assess water resources^21,22^.

**Figure 1:**
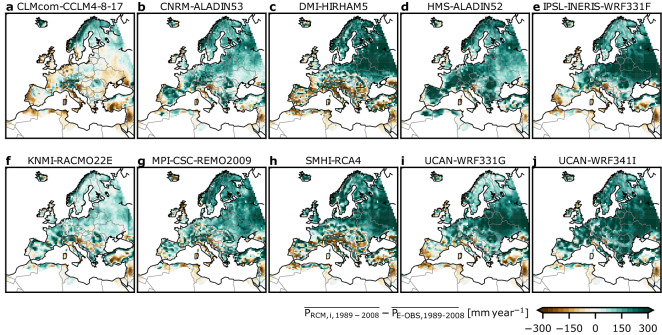
Example for precipitation biases. Differences of long-term mean (1989 to 2008) annual sums of the individual EURO-CORDEX ensemble RCMs used in this study and E-OBS gridded observations [mm year^−1^]. A common mask is applied to ensure a spatial overlap between RCMs and E-OBS. Data is shown on the native EURO-CORDEX 0.44° degree resolution EUR-44 grid. The Med-CORDEX model domain (not shown) encompasses the Mediterranean area from about 10 W to 50 E and 25 N to 60 N in the Southern part of the pan-European EURO-CORDEX domain.

Despite continuous progress over the years^4^, RCMs often show precipitation biases over land areas, that significantly affect the water cycle (Fig. 1). This behaviour has been documented over the course of multi-model ensemble projects, such as PRUDENCE^23,24^, ENSEMBLES^25^, or NARCCAP^26^. In the more recent EURO-CORDEX RCM ensemble^27^ driven by ERA-Interim reanalysis, a standard evaluation over Europe shows a wide range of precipitation biases across the different members. Seasonal and sub-domain biases range between − 40 and + 80% with a tendency in the long-term mean spatial bias patterns to overestimate precipitation over the central, northern, and eastern parts of the model domain and along the eastern model boundary, and an underestimation over the southern parts of Europe^28^. In addition, the observed biases exhibit seasonal and regional variability^29^, just recently demonstrated in a study evaluating EURO-CORDEX ensemble members together with high-resolution GCM outputs^30^. In RCMs, the impact of precipitation biases becomes also apparent in large-scale studies that assess all components of regional water budgets over large European catchments^31^. Therefore, in general, hydrology-related climate change impact studies, as mentioned above, often rely on precipitation bias-adjusted RCM simulation results^32,33^.

The RCM-simulated precipitation characteristics may be attributed to a number of factors including model resolution^34–37^, convection^36,37^ and microphysics schemes^37,38^, and aerosol treatment^39^. In addition, evaporation and evapotranspiration, calculated by the RCMs' surface and atmospheric boundary layer schemes affect the atmospheric water budgets and indirectly precipitation through land–atmosphere feedback processes^40,41^, precipitation recycling^42^, and moisture transport^43^. Furthermore, Refs.^44–46^ analysed the influence of sea surface temperatures (SSTs) on precipitation also over land areas. In Ref.^46^, corrected SST biases from the driving GCM led to circulation and atmospheric moisture transport changes in the RCM’s water cycle and eventually to a reduced wet bias over continental areas of southern Africa. An increased evaporation over the Mediterranean Sea surface due to increased SSTs in Ref.^45^ can lead to more extreme precipitation events during summer in Central Europe in association with cyclonic activity; a strong link also exists between Mediterranean SST and precipitation over the Anatolian Peninsula^47^. Reference^44^ show in a climate change ensemble over the Baltic for some RCMs a positive correlation between SST and widespread precipitation.

**Figure 2:**
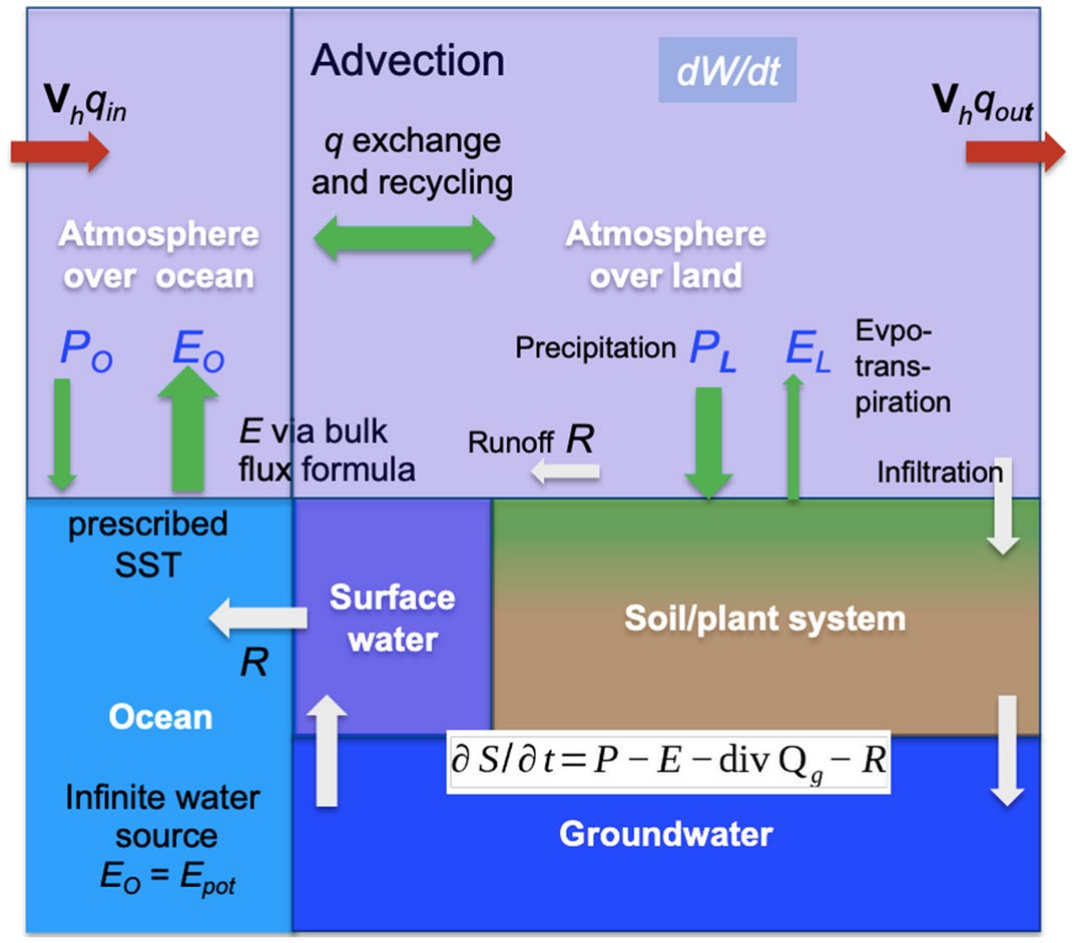
Schematic of main water cycle components and fluxes as used in this study. The variables are explained in the main text. The width of the arrows is not in exact proportion to each other, it shall indicate the relative magnitude of the fluxes.

In this study, we re-assess the atmospheric compartment of the hydrological cycle of RCMs^48–50^, as a driver of the of the terrestrial water budget. We follow theoretical water budget considerations applied previously^31,43,51,52^, where the simplified time-averaged general balance equation for total water in an atmospheric column is defined as^53^

1 $$\partial W/\partial t+{\text{div}} \, {\mathbf{Q}}=E-P$$

where *W* is the total column water content (in its liquid phase as precipitable water, as water vapor, and as ice), *P* is total precipitation (including snow fall), *E* is evaporation over the oceans and evapotranspiration over land. The atmospheric moisture divergence div **Q** is the vertically integrated horizontal moisture transport, defined as

2 $${\text{div}} \, {\mathbf{Q}}=-\nabla \cdot 1/g{\int }_{0}^{{p}_{s}}q{\mathbf{V}}_{h}dp$$

with the gravitational acceleration *g*, the specific humidity *q*, the horizontal wind vector **V**_*h*_, pressure *p* and surface pressure *p*_*s*_. Figure 2 gives a schematic overview of the water cycle components and fluxes, from the groundwater to the atmosphere. Because the atmospheric water storage change, i.e., the tendency term in Eq. (1), is over long time scales (here: d*t* is 1 year) negligible,

3 $$\partial W/\partial t\approx 0$$

the primary balance is between the atmospheric moisture divergence div **Q** and *E* and *P*. Thus, the atmospheric moisture divergence div **Q** is driven by *E* as the source and *P* as the sink. Hence a simplified atmospheric water budget or balance for a control volume of the RCM model domain, i.e., all land–ocean grid points, the land, and the ocean areas can be expressed as

4 $${\text{div}} \, {\mathbf{Q}}=E-P$$

A net export of atmospheric water is expressed by a positive divergence, and vice versa.

Because an RCM as a limited area model (LAM) numerically constitutes a boundary value problem, the atmospheric moisture divergence div **Q** of the complete model domain is constrained by lateral advection of the boundary forcing fields *q*_*in*_ and *q*_*out*_

5 $${\text{div}} \, {\mathbf{Q}}\approx {q}_{in}{\mathbf{V}}_{h,in}-{q}_{out}{\mathbf{V}}_{h,out}$$

from the driving model, such as a GCM. After initialisation, the RCM is driven by regularly updated, temporally and spatially interpolated meteorological boundary conditions for most of the prognostic variables. This is the fundamental property of a LAM. The procedure to ingest the forcing data into the RCM is still an issue that requires critical attention^4,5^. Based commonly on the theory described in Ref.^54^, a relaxation term is added within the lateral boundary relaxation zone to the prognostic equations, constraining the model to the boundary conditions^55^. Alternatively, less constrained boundary condition schemes than the Davies relaxation also exist, such as the Mesinger scheme^56^. In the reanalysis-driven coordinated RCM ensemble used in this study, all RCMs apply the same lateral boundary conditions based however on varying implementations of the relaxation in the different models of the ensemble.

Through *P* and *E*, the atmospheric water budget is directly coupled with the terrestrial water budget, including groundwater

6 $$\partial S/\partial t=P-E-{\text{div}}{ \, {\mathbf{Q}}}_{g}-R$$

where *S* is the total terrestrial water storage, *R* is runoff, and div **Q**_*g*_ is groundwater divergence^52^.

The evapotranspiration over land, *E*_*L*_*,* and evaporation over the ocean, *E*_*O*_, is calculated based on bulk flux algorithms, using implementations of the Monin–Obukhov similarity theory framework^57^. Over water surfaces the turbulent vertical fluxes are calculated by the model’s surface layer scheme, primarily controlled by the SSTs, friction velocities, and moisture, heat and momentum transfer coefficients. Over land, the friction velocities and transfer coefficients may be passed from the surface layer scheme to the land surface model to calculate the vertical fluxes. The surface scheme parameterizations differ between models and are very sensitive to the transfer coefficients, which in turn depend on the Monin–Obukhov universal functions and flux-profile relationships. It has been shown in model comparisons with forced SSTs that there are differences in *E*_*O*_^58^ and that RCMs^58,59^ tend to overestimate the latent heat flux over the oceans due to varying reasons^59,60^.

Using a simplified atmospheric water budget definition as given in Eq. (4), the goal of this study is to characterise the connection between the atmospheric water budget components over land and ocean in the constrained setup of an RCM ensemble, taken here from the CORDEX project, with prescribed div **Q** through the same reanalysis ERA-Interim boundary conditions. This constrained setup affords an assessment of the RCMs in maintaining the imposed global model's water budget and redistributing moisture within the model domain. While individual RCM results are not relevant in the characterisation, a model run identification is used to support the interpretation of results in comparison with evaluation studies such as Refs.^28,29,61^ without the intention of pursuing a validation study or a detailed assessment of the individual behaviours of the RCMs. Through a conceptual budget study based on annual data, the goal is rather to re-assess fundamental atmospheric water source-sink relationships in a LAM in relation to the superimposed moisture flux divergence of the driving model along the boundaries.

The analyses show that in a state-of-the-art RCM ensemble over Europe using prescribed SSTs, the combination of the RCMs' numerical property of being a boundary value problem with excess oceanic evaporation contributes to the often-seen positive precipitation bias over land. The RCMs model solution is relaxed to the superimposed driving model’s atmospheric moisture flux divergence, as prescribed through the lateral boundary conditions. As a result, excess atmospheric moisture is recycled through precipitation over land.

## Results

### Annual atmospheric water budgets

**Figure 3:**
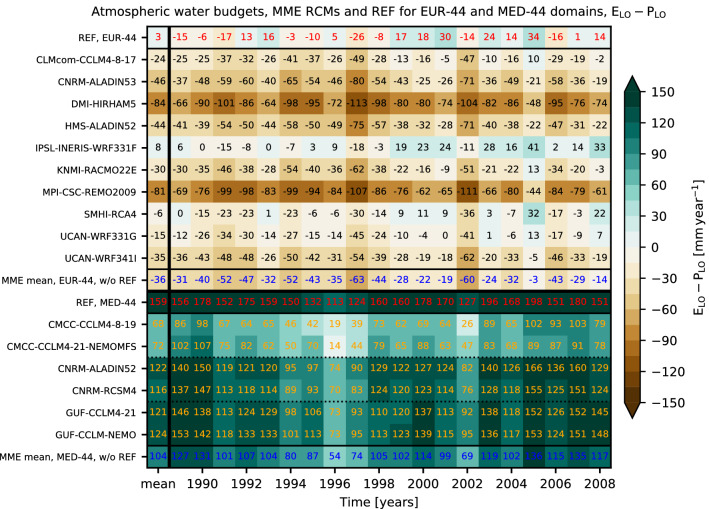
Atmospheric water budgets (div **Q**_*LO*_ = *E*_*LO*_–*P*_*LO*_ [mm year^−1^]) from 1989 to 2008 from domain-averaged annual *P*_*LO*_ and *E*_*LO*_ sums. Upper part: Pan-European domain (EUR-44); lower part: Mediterranean domain (MED-44), the stippled lines separate the different RCM/AORCM model pairs. First row of each part: REF (red numbers); subsequent rows below: each RCM (black and yellow numbers); last row of each part: multi-model mean (blue numbers). The leftmost column shows the 20-year div **Q**_*LO*_ averages of REF and each RCM. The REF data are remapped to the 0.44° grid. The 20-year spatial averages for REF are P_REF EUR44_ = 643 mm year^−1^ and E _REF EUR44_ = 640 mm year^−1^ for the pan-European domain and P_REF MED44_ = 516 mm year^−1^ and E _REF MED44_ = 675 mm year^−1^ for the Mediterranean domain.

Figure 3 shows the annual atmospheric water budgets of the 10-member multi model ensemble (MME) of EURO-CORDEX RCMs and an additional 6-member subset of the Med-CORDEX ensemble^62^ with three uncoupled (RCM) and coupled (Atmosphere–Ocean–RCM, AORCM) model pairs of CORDEX evaluation runs, in comparison to the ERA-Interim forcing (REF). The budgets are calculated as spatial means of all land–ocean grid points of the annual sums of *E*_*LO*_ and *P*_*LO*_ [mm year^−1^] based on Eq. (4) over the complete EURO-CORDEX 0.44° (Fig. 1) pan-European and Med-CORDEX 0.44° Mediterranean focus domains for each year of the official CORDEX 20-year evaluation time span from 1989 to 2008.

**Figure 4:**
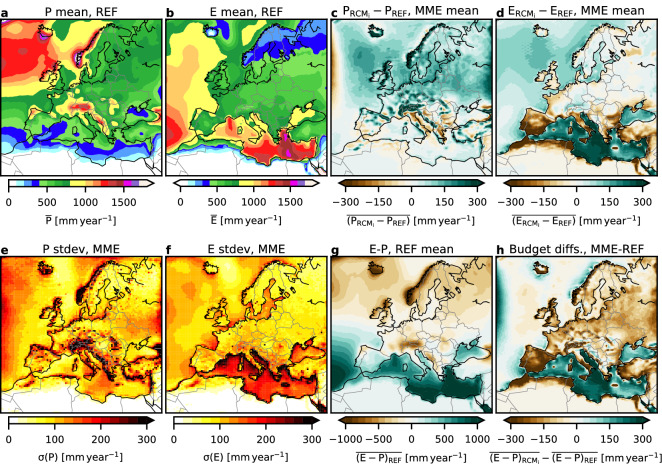
REF long-term mean (1989 to 2008) annual sums of (**a**) *P* and (**b**) *E* [mm year^−1^]; MME mean of individual RCM long-term mean annual sums minus REF of (**c**) *P* and (**d**) *E* [mm year^−1^]. MME standard deviation of the individual RCM long-term mean annual sums of (**e**) *P* and (**f**) *E* [mm year^−1^]. (**g**) Long-term mean atmospheric water budget (*E*–*P*) per grid cell for REF. (**h**) Difference of atmospheric water budgets, MME mean of the individual long-term mean budgets (*E*–*P*) of the RCMs minus the long-term mean budgets (*E*–*P*) of REF (see subplot **g**). Data is shown on the native EURO-CORDEX 0.44° degree resolution EUR-44 grid. Note the Med-CORDEX data are not shown in this plot.

Because the complete model domains are considered (Figs. 1, 4), the atmospheric water budgets correspond to the overall moisture flux divergence div **Q**_*LO*_ of the limited area models. As the atmospheric water storage change can be considered negligible over longer time spans (Eq. 3), div **Q**_*LO*_ is expected to match the sink and source terms that are *P*_*LO*_ and *E*_*LO*_. Because the MME members are all forced by similar lateral atmospheric boundary conditions and SSTs from REF (in case of the uncoupled RCMs with prescribed SSTs), the overall MME budgets should match closely div **Q**_*LO*_ of REF in the long-term means of the annual sums.

Note, the REF atmospheric budget and *P*_*REF*_ and *E*_*REF*_ are not to be understood as quantities against which the MME members are validated; REF is indeed a reference data set for the MME members since they are based on the REF forcing along the boundaries (and over the oceans in case of prescribed SSTs) in the RCM downscaling approach.

The boundary forcings may vary between individual RCMs due to differences in the configuration of the retrieval of the ERA-Interim data by different RCM modelling groups, differing RCM model grids, spatial interpolation settings, and boundary relaxation procedures. Thus, each RCM is constrained to potentially different boundary conditions, while resembling the same large-scale features of the driving REF forcing fields, which may result in differences in global and local water budget estimates. However, these differences are supposed to be small relative to the overall budgets.

The REF atmospheric water budget (Fig. 3, “REF, EUR-44”) for the pan-European model domain of EURO-CORDEX is almost divergence-free with a small positive long-term mean budget where *E*_*LO*_ exceeds *P*_*LO*_ by about 3 mm year^−1^. The interannual variability reflects the general circulation of the atmosphere. In a year such as 1996 with div **Q**_*LO*_ ≈ 5 mm year^−1^, i.e., *P*_*LO*_ ≈ *E*_*LO*_, the lateral import is close the export of atmospheric water for the pan-European EUR-44 control volume under consideration. The fact that reanalyses such as REF are based on atmospheric water mass conservation constraints due to the analysis increments^43^ is not relevant, because of the rationale that over the model domain, all MME members are constrained laterally by the same div **Q**_*LO*_ of REF, which has to be matched in the simulations. However, in case of the pan-European MME under consideration, negative atmospheric water budgets (i.e., *P*_*LO*_ > *E*_*LO*_) dominate (9 out of 10 RCMs), with an average of div **Q**_*LO*_ = − 36 mm year^−1^ over a 20-year period, despite the fact that all RCMs are supposedly driven by more or less the same atmospheric moisture and SST boundary conditions from REF. The obvious question is, where does the excess water originate from in the pan-European MME simulations?

In stark contrast, for the Mediterranean domain of Med-CORDEX (Fig. 3, “REF, MED-44”), the long-term atmospheric REF water budget is clearly characterized by a positive divergence, i.e., a net export of moisture, reflecting a source region where *E*_*LO*_ exceeds *P*_*LO*_ by about 159 mm year^−1^ on average. For the Mediterranean MME, all RCMs show a positive atmospheric water budget following REF, albeit with an average of a div **Q**_*LO*_ = 104 mm year^−1^.

### Role of the ocean areas

The spatial patterns of *E* and *P* in Fig. 4 provide insight in the RCMs' systematic behaviour shown in Fig. 3; for brevity only shown for the pan-European model domain based on the EURO-CORDEX MME. The REF dataset in Fig. 4a,b shows typical long-term mean spatial patterns with regional *P* maxima associated with the Icelandic Low, orographic precipitation along the Norwegian coast, the Alps and Scotland, and declining precipitation in Northern Africa. The *E* distribution is characterized by a sharp contrast between ocean and land areas, and areas of high *E* associated with the North Atlantic Current, the Azores High and the Eastern Mediterranean.

The spatial difference plot between the MME mean *P* and the REF data in Fig. 4c is characterized by generally higher MME precipitation in the Northern part of the model domain. The largest differences appear along mountain ranges, e.g., Norway, German mid mountain ranges, and the Pyrenees. In the 0.44° resolution RCMs, with stronger relief compared to the 0.75° resolution REF, more orographic rainfall and less rainfall in the adjacent lowlands is induced due to smoothing effects.

Assuming a mean zonal atmospheric water transport with the prevailing westerly large-scale flow, the MME has a spinup (relaxation) zone along their westernmost boundary with less average precipitation than the global REF data (Fig. 4c), because the lateral forcing in the RCMs has to be picked up, e.g., by the microphysics and convection schemes before precipitation can be generated. Along the easternmost RCM boundary relaxation zone, where the main outflow from the RCMs takes place, an area with a maximum *P* difference indicates that the RCMs' atmospheric advection is relaxed against the lateral boundary conditions of the REF, which causes some of the excess precipitation in the RCMs. This is the area where eight out of nine RCMs in the EURO-CORDEX evaluation study of Ref.^28^ (their Fig. 4) show strong precipitation biases during summer. The inter-model spread in *P* in the MME shown in Fig. 4e is also highest along the western and eastern relaxation zones. Orographic features and coastlines are also characterized by a larger spread, most likely due to differences in RCM model configurations, such as slight changes in the land–ocean masks or differences in the underlying orography used by the RCM.

The difference of *E* in Fig. 4d is indicative of larger values in the MME than in REF over nearly all ocean areas with a pronounced maximum difference in the Mediterranean, which is in line with an evaluation study of Mediterranean Sea water and heat budgets^58^ and the *E* and *P* relationships in the eastern Mediterranean^63^. Differences between MME and REF over land are relatively small and might be attributed to differences in the landmask (close to the coast), landuse datasets (see, e.g., Southern Spain), or the RCMs’ land surface model. Also, over land, the evapotranspiration is energy- or water-limited and controlled by vegetation^64^, indicated by the lower standard deviation of *E* of the MME in Fig. 4f over land as opposed to precipitation in Fig. 4e. However, over the ocean areas, especially the Mediterranean, the standard deviation σ(*E*_*O*_) is very high, indicating a large MME spread and highly differing long-term mean latent heat fluxes, despite the fact that all MME members use the same prescribed SSTs from REF. This behaviour has been attributed to the choice of the surface layer scheme in combination with roughness length, exchange coefficients, and the stability of the boundary layer, model resolution, as well forced SST versus coupled ocean–atmosphere modelling approaches^47,58–60^.

Figure 4g gives insight in the spatial distribution of source and sink areas of the atmospheric water balance. Independent of lateral in- or outflow of atmospheric moisture, the Mediterranean and southern parts of the Eastern Atlantic with the Azores High are net source areas of atmospheric moisture while continental areas act as net sinks, which is well-known^53,58,63,65^. In case of ocean areas, the difference of the *E*_*O*_–*P*_*O*_ atmospheric water balances of MME and REF (Fig. 4h) reveals the largest differences over the Mediterranean due to a higher MME *E*_*O*_ between 200 and 300 mm year^−1^ on average than in REF (Fig. 4d). The differences between the REF and MME atmospheric water balances over the Atlantic are smaller (Fig. 4h). Over land areas in Fig. 4h the continental atmospheric moisture sink is stronger in the MME *E*_*L*_–*P*_*L*_ budget than in the REF budget, due to a higher *P*_*L*_ in the RCMs (Fig. 4c).

### Boundary condition effects and the connection of atmospheric water budgets over land and ocean—the origin of excess water

**Figure 5:**
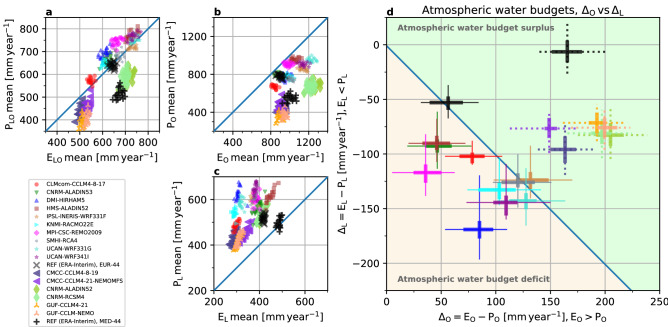
Relationship of *E* versus *P* for (**a**) all grid points, (**b**) ocean grid points, (**c**) land grid points; spatial means of annual sums for 20 years from 1989 to 2008 for the EURO-CORDEX RCMs (upper 10 coloured symbols) and accompanying REF data (black cross) and the Med-CORDEX RCMs and AORCMs (lower 6 coloured symbols) and accompanying REF data (black plus sign); note the different data ranges among the individual plots. (**d**) Atmospheric water budget over the ocean (x-axis) versus over land (y-axis); EURO-CORDEX models: solid lines, Med-CORDEX models: stippled lines; due to differences in the areal fraction of land and ocean grid elements, the spatial means of the annual sums per RCM and REF are weighted with the respective number ocean and land grid points; whiskers as thin horizontal and vertical lines along Δ_*O*_ and Δ_*L*_ denote minimum and maximum, their intersection is the Δ_*O*_ and Δ_*L*_ arithmetic mean; the boxes as thick horizontal and vertical lines show the interquartile range. The same symbols are used for the same RCM family; each simulation uses a different model configuration. For legibility the individual annual realisations per model from sub-figure (**a**,**b**, **c**) are not shown in sub-figure (**d**); the colour-coding is retained for each model.

As the ocean areas play an important role in the RCMs' atmospheric water budgets, Fig. 5 separates the atmospheric water budget contributions of individual RCMs and REF on an annual and long-term basis for the EURO-CORDEX and Med-CORDEX models; the behaviour of the latter is described at the end.

In Fig. 5a, *E*_*LO*_ versus *P*_*LO*_ values are plotted based on all grid points. Apart from interannual variability, REF for the pan-European model domain has an almost divergence-free atmospheric water budget consistent with Fig. 3, where div **Q**_*LO*_ is calculated based on the same value pairs. As expected, for ocean areas, *E*_*O*_ > *P*_*O*_ in Fig. 5b results in excess water in the atmosphere over the oceans. This leads to a characteristic *E*_*L*_ < *P*_*L*_ relationship over land (Fig. 5c), where the excess *P*_*L*_ may generate runoff, and changes in the sub-surface storage and the div **Q**_*g*_ term (Eq. 6), based on the parameterizations inherent in the different land surface schemes^52,53,65^. Considering the MME, the hydrological cycle is more intense for most RCMs compared to REF, with a higher *E*_*LO*_ and *P*_*LO*_ than REF (Fig. 5a), always constrained with the moisture inflow and outflow along the RCM boundaries as well as the SST. When comparing Fig. 5b,c, the relationship is basically inverted. With an infinite water source over the oceans, *E*_*O*_ is literally unconstrained and close to the potential evaporation, *E*_*pot*_. Over land, *E*_*L*_ is limited^64^, here *P*_*L*_ shows a large spread, whose range resembles approximately the spread of *E*_*O*_.

Figure 5d relates the respective atmospheric water budgets over the oceans on the x-axis, Δ_*O*_ = *E*_*O*_–*P*_*O*_, to the atmospheric water budgets over land on the y-axis, Δ_*L*_ = *E*_*L*_–*P*_*L*_. For completeness, measures of the statistical distribution of the 20-year sample per MME member and REF are shown. The relationship between Δ_O_ and Δ_L_ resembles the mass flux between the ocean and land areas with excess evaporation (*E*_*O*_ > *P*_*O*_) over the oceans and excess precipitation (*E*_*L*_ < *P*_*L*_) over land. Because the lateral transport into and out of the RCM domain across the boundaries is fixed through the boundary conditions from REF, the MME members should in principle closely follow the REF Δ_*O*_ and Δ_*L*_ values.

In Fig. 5d, REF reflects the prescribed divergence over the limited area implemented as boundary conditions in the different RCMs, which is on average close to zero for the pan-European model domain. The areas above and below the 1:1 line are characterized by an atmospheric water budget surplus or deficit in the RCM, respectively (see also Fig. 3), which can only stem from the prescribed boundary condition assuming that the model accurately conserves moisture and energy. Any shift away from REF and parallel to the 1:1 line means varying partitioning of Δ_O_ and Δ_L_ because of different physics, dynamics, parameterisations, and internal model variability, while maintaining the overall mass balance over the model domain enforced by the REF boundary forcing. In contrast, any shift away from REF and perpendicular to the 1:1 line means that the MME member’s boundary forcing differs from REF assuming mass conservation in the numerical implementation of the boundary value problem.

Interestingly, the 20-year means, of the individual RCMs show a clear tendency with nine out of ten falling below the 1:1 line, suggesting a negative divergence over the model domain enforced by the boundary condition, resulting in a surplus of precipitation over land areas (see Fig. 1). Thus, it appears that there are major differences between RCMs in the implementation of the same boundary condition of REF changing the model domain from being divergence-free to a net sink. In addition, means are shifted parallel to the 1:1 line due to an *E*_*O*_ increase in comparison to REF (see Fig. 5b), leading to an additional contribution to the positive *P*_*L*_ bias (see Fig. 1). This is the oceanic impact and is especially apparent in the results of individual RCMs that fall close to the 1:1 line. Thus, we identify differences in the implementation of the prescribed boundary conditions and increased oceanic evaporation as important factors in an intensified ocean-land recycling rate, that contributes to the positive precipitation bias in RCMs.

In comparison, the Med-CORDEX models are characterized by an atmospheric water budget surplus (see Fig. 3) and thus a positive divergence over the Mediterranean model domain. In Fig. 5d those RCMs follow REF in that all results fall above the 1:1 line, making the Mediterranean domain act as a net exporter of moisture, which is in contrast to the results for the pan-European model domain. The net export is however less pronounced in case of the RCMs illustrating again the differences in the implementation of the boundary conditions in the different models (a shift away from REF perpendicular to the 1:1 line). In addition, the RCM results are shifted parallel to the 1:1 line in the direction of an increased oceanic evaporation; in one of the coupled AORCMs this effect is clearly weaker than in the uncoupled counterpart.

### The impact of the land on the RCMs’ deviations from the REF budget

To further assess how the MME water budgets deviate from REF as shown in Fig. 5d, we relate *P* and *E* from MME to REF. Following our rationale, the MME member should match the driving atmospheric water budget of REF

7 $$-{\text{div}} \, {\mathbf{Q}}_{RCMi}+{\text{M}}_{RCMi}+{\left(E-P\right)}_{O,RCMi}+{\left(E-P\right)}_{L,RCMi}=-{\text{div}} \, {\mathbf{Q}}_{REF}+{\text{M}}_{REF}+{(E-P)}_{O,REF}+{(E-P)}_{L,REF}$$

where *M* represents all atmospheric moisture increments related to potential mass conservation continuity violations in the numerical implementation of the individual models; and div **Q**_*LO*_ are the divergences that are prescribed by the boundary conditions and SSTs in case of the RCMs. In this way, all mass balance deviations and variations in the superimposed div **Q**_*LO*_, with varying mass import and export, can be accounted for conceptually.

To assess, which component, *P* or *E*, contributes to the overall atmospheric water budget, differences among REF and MME are calculated

8 $$\Delta M + \Delta {\text{div}}\;{\mathbf{Q}} = \Delta E_{O} - \Delta P_{O} + \Delta E_{L} - \Delta P_{L}$$

**Figure 6:**
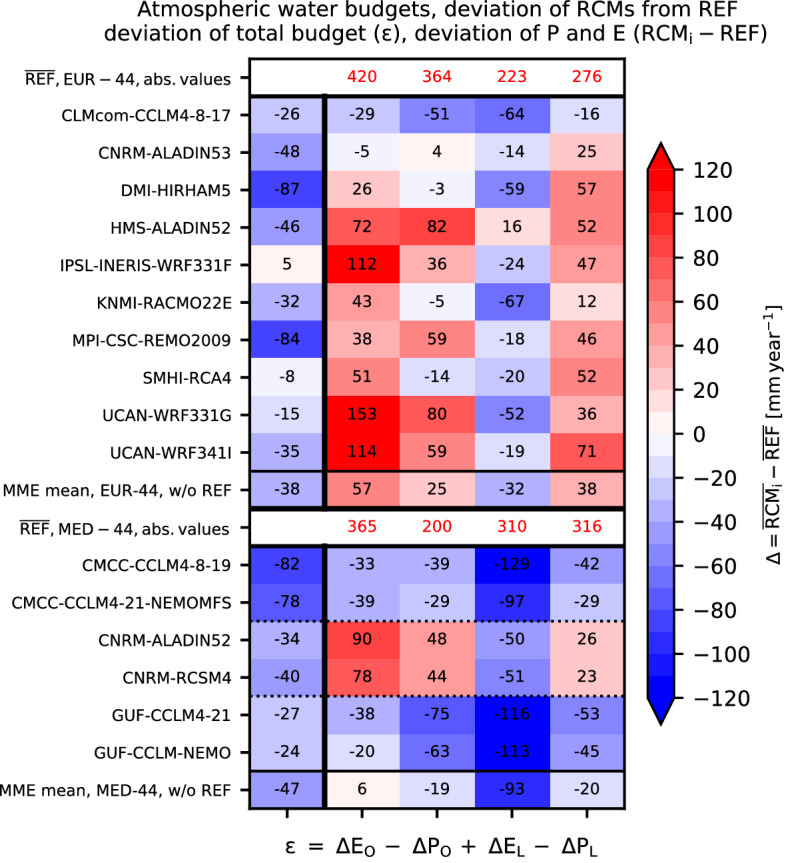
Contribution of atmospheric water budget components to the individual MME member deviation from REF. Upper rows: Pan-European domain; lower rows: Mediterranean domain, the stippled lines separate the different RCM/AORCM model pairs. Columns resemble the terms of Eq. (8), see the x-axis labels. Colours indicate the deviations (RCM_i_ − REF), calculated from 20-year mean annual sums. The upper and lower white rows contain the long-term means of the REF for *E*_*O*_, *P*_*O*_, *E*_*L*_, and *P*_*L*_ for the pan-European and Mediterranean model domains, respectively. An area weighing accounts for different land–ocean fractions (using the REFs’ land–ocean distribution).

With $\Delta = RCM_{i} - REF$. Because this simplified mass budget framework does neither allow nor intend to determine the relative contributions of Δ*M* and Δdiv **Q**, these are summed in the increment ε = Δ*M* + Δdiv **Q**. Figure 6 shows the estimates of the different increments for REF and the RCMs for the pan-European and Mediterranean regions.

All but one pan-European RCMs show negative *ε* values (Fig. 6), i.e., they are net sinks and, thus, plot below the 1:1 line in Fig. 5d. In addition, the estimates of an increased *E*_*O*_ and *P*_*L*_ confirm the enhanced recycling rate that is identified as one of the factors for a positive precipitation bias in the RCMs; eight out of ten pan-European RCMs show this behaviour. The elevated *E*_*O*_ leads also elevated *P*_*O*_, i.e., an enhanced recycling over the ocean, but there is a net transport onto the land areas. The individual models show a diverse behaviour with respect to the magnitude of their deviation from REF. What is generally apparent, however, is the low *E*_*L*_ of many models in comparison to REF, which is another factor in the negative *ε* as described in Fig. 5d.

In Fig. 5d, pan-European models left of REF deviate either very little over the ocean from REF (CNRM-ALADIN53) or show a very strong recycling (HMS-ALADIN52). The Fig. 5d ocean-land atmospheric water budget relationship far off the 1:1 line of DMI-HIRHAM for example, is according to Fig. 6 mainly due to a combination of enhanced *P*_*L*_ with low *E*_*L*_. The one model which is on average a moisture source and close to the 1:1 line in Fig. 5d (IPSL-INERIS-WRF331F) exhibits a higher *E*_*O*_ than REF with enhanced recycling over water, but also a smaller than average *E*_*L*_ deviation. At the same time, six out of ten pan-European RCMs, which show an above-average Δ*P*_*L*_ as compared to REF in Fig. 6, also show high positive precipitation biases in Fig. 1; in three cases this is associated also with above-average *E*_*O*_ and low *E*_*L*_ deviations (HMS-ALADIN52, IPSL-INERIS-WRF331F, UCAN-WRF341I).

The three Mediterranean model pairs (two times CCLM, one ALADIN atmospheric model) follow the pattern of their pan-European RCMs counterparts. In the AORCM coupled model versions the *E*_*O*_ is thereby slightly reduced (Δ*E*_*O*_ in Fig. 6). In general, the RCMs’ and AORCMs’ atmospheric divergence is smaller than REF for the Mediterranean.

## Discussion

Based on results from a EURO-CORDEX MME for a pan-European model domain, supplemented by a smaller Med-CORDEX MME for a Mediterranean model domain, our analysis indicates a clear deviation of individual RCMs of the integrated annual and long-term mean atmospheric water budgets from the driving REF ERA-Interim reanalysis. In other words, in the RCMs, differences in the implementation of the boundary conditions lead to differences in div **Q**_*LO*_ between the RCMs and REF. Assuming that the RCMs are mass conserving, the boundary conditions will force the models’ sink and source terms to balance the REF divergence via coupling of the atmosphere with the ocean and land surfaces. Thus, the prescribed divergence along the RCMs’ boundary effectively determines the overall fluxes in the domain. Understanding the technical details of the implementation of the boundary forcing in the different models is clearly beyond the scope of this study. One potential reason may be that in the boundary relaxation, specific humidity and the velocities are treated individually. In addition, most RCMs show an intensified ocean-land moisture recycling that is systematic, overestimating *E*_*O*_ compared to *E*_*O,REF*_ and an excess *P*_*L*_.

From these RCM behaviours, we conclude that the differences in div **Q**_*LO*_ in conjunction with excess *E*_*O*_ contribute significantly to the positive *P*_*L*_ biases seen in evaluation studies of RCMs^24,28,30,66^, because eventually the imposed atmospheric moisture flux divergence from the lateral boundary conditions of the driving global ERA-Interim reanalysis, must be met. Hence, the combination of boundary-imposed atmospheric moisture transport, oceanic surface layer fluxes, and recycling leads to biases in reproducing the observed terrestrial precipitation and the terrestrial water cycle. This behaviour is for some models more pronounced than for others.

The RCM and AORCM simulations of Med-CORDEX over the Mediterranean are all, in terms of the atmospheric water budget, net sources with a strong positive divergence over the model domain. In general, a similar behaviour can be observed as with the pan-European model runs.

With respect to the *E*_*O*_, model comparison studies with prescribed SSTs indicated differences in *E*_*O*_^58^ and tendencies of RCMs to overestimate the latent heat flux over the oceans^58,59^. Reasons for the deviation from observations are overestimated surface exchange coefficients associated with high surface roughness in surface schemes; atmospheric stability associated with radiation schemes, that affect the surface energy balance and thereby coupling and fluxes^59^; and atmospheric model horizontal resolution leading to different wind speed regimes and resulting turbulent exchange fluxes. Also, interactively coupled ocean–atmosphere models may lead to more realistic SSTs than RCM simulations with prescribed forced ocean SSTs^60^, which also affects precipitation over land areas^44–46^. The AORCMs with a coupled dynamic ocean used in this study also show a tendency, with two out of three model pairs, towards a reduction of the excessive *E*_*O*_ seen in RCMs with prescribed SSTs.

The rationale of this study is founded on the basic atmospheric water mass conservation consideration, that is div **Q** = *E*–*P* must hold over long time scales. Because div **Q** is prescribed in a LAM, any changes in the source term must be balanced by changes in the sink term and vice versa, given that the numerical implementation follows mass conservation. This is independent of model resolution^34–37,67^, convection^36,37^ and microphysics schemes^37,38^, aerosol treatment^39^, land–atmosphere feedback processes^40,41^ or precipitation recycling^42^. These factors determine internal model behaviour and variability and come into play in studies of spatiotemporal patterns of fluxes and states of water cycle components, and local water budgets.

Our study expands these cause-and-effect relationships that are required in the explanation of the precipitation biases in RCMs. In summary, the driving model's superimposed moisture flux divergence, excess oceanic evaporation in RCMs with prescribed SSTs, and the recycling of moisture have a combined impact on terrestrial precipitation biases and water budgets. The results suggest a need for careful re-inspection of the RCMs' implementation of the boundary conditions and surface schemes, in conjunction with forced SSTs. In this context, Big Brother-type experiments could be used to assess the impacts, e.g., of the lateral boundary condition implementations^68^ in the experiment preparation. These measures will help in reducing differences in the atmospheric water budgets and likely reduce excess *E*_*O*_ in the RCMs and *P*_*L*_ biases. The two major ongoing developments in regional climate modelling, towards convection-permitting spatial resolutions and coupled (particular ocean–atmosphere) regional Earth system models^4,5^, already address some of the sources of precipitation biases.

## Methods

### Model data

The RCM simulation results are from the World Climate Research Programme's (WCRP) Coordinated Regional Downscaling Experiment (CORDEX) project, a diagnostic model intercomparison project for CMIP6^9^. Data from two initiatives are used: EURO-CORDEX, for a pan-European model domain, is the main dataset under investigation; a supplementary smaller dataset is used from the Med-CORDEX initiative for a Mediterranean domain.

The EURO-CORDEX data is available through the Earth System Grid Federation (ESGF) data dissemination system^69^. Based on data availability on ESGF, ten RCM ensemble members from the EURO-CORDEX initiative, the European branch of the CORDEX project, are used. Important for this study, RCM data are from the EURO-CORDEX evaluation experiment^27,28^, i.e., the 3- to 6-hourly lateral boundary forcing and the prescribed daily SST, are from the ECMWF ERA-Interim reanalysis^70^ and therefore similar for each ensemble member (driving experiment meta data: "ECMWF-ERAINT, evaluation, r1i1p1"). The following RCMs are used, identified by their unambiguous CORDEX experiment protocol institute ID, and the RCM model and version IDs: CLMcom/CCLM4-8-17/v1; DMI/HIRHAM5/v1; IPSL-INERIS/WRF331F/v1; KNMI/RACMO22E/v1; MPI-CSC/REMO2009/v1; SMHI/RCA4/v1; UCAN/WRF331G/v1; UCAN/WRF341I/v1; CNRM/r1i1p1/ALADIN53/v1; HMS/r1i1p1/ALADIN52/v1. The RCMs have been extensively validated and described in their CORDEX configurations^28,29,61^. RCM data are used on a CORDEX-defined rotated latitude–longitude EUR-44 grid at 0.44° horizontal resolution, with a 106 × 103 grid points focus domain. This EUR-44 grid defines the spatial reference throughout the study (Fig. 3). The boundary relaxation zone, which differs per RCM, depending on the individual dynamical downscaling setup, is removed before simulation results are checked for compliancy (CMOR standard) and ingested into the ESGF. Because the ALADIN RCMs use a different model grid, a 1st order conservative remapping to the EUR-44 grid with the Climate Data Operators (cdo) (v1.9.1)^71^, is applied. Variables used are precipitation [kg m^−2^ s^−1^] ("pr"), defined to include both liquid and solid phases from large-scale and convective clouds, and surface evaporation [kg m^−2^ s^−1^] ("evspsbl"), defined as the flux of water into the atmosphere due to conversion of both liquid and solid phases to vapor (from underlying surface and vegetation), at a daily resolution, available for the official EURO-CORDEX evaluation time span from 1989 to 2008, stored in netCDF files at 5-year intervals. For each RCM the respective landmask [0–100%] ("sftlf") is available.

The Med-CORDEX initiative^62^ data are from the phase 1 core and tier 1 simulations, available through the Med-CORDEX data dissemination infrastructure (https://www.medcordex.eu, THREDDS server). The same variables as for EURO-CORDEX are used, albeit the base data retrieved are at a monthly temporal resolution. For compatibility, Med-CORDEX simulations at 0.44° horizontal grid resolution, on a 98 × 63 grid point focus domain (MED-44) are selected. This grid specification nearly exactly overlaps with the EUR-44 grid. Specific to the Med-CORDEX initiative experiment is the availability of coupled Regional Climate System Models (RCSM) or Atmosphere–Ocean Regional Climate Models (AORCM), that feature, e.g., fully interactive ocean model components, which cover the whole Mediterranean, usually at a higher resolution than the atmospheric model components. The subset of models used here has been selected as there is a coupled AORCM counterpart to each uncoupled RCM with prescribed SSTs. The following subset of three model pairs of Med-CORDEX models was available that matched the aforementioned criteria: CMCC/CCLM4-8-19/v2 (RCM, SST update daily from ERA-Interim) with CMCC/CCLM4-21-NEMOMFS/v1 (AORCM); CNRM/ALADIN52/v1 (RCM, SST update monthly from ERA-Interim) with CNRM/RCSM4 v1 (AORCM); GUF/CCLM4-21/v1 (RCM, SST update daily from ERA-Interim) with GUF/CCLM-NEMO/v1 (AORCM). Due to the complex model setups of the coupled AORCMs, model configurations are more heterogeneous than with EURO-CORDEX; for example, the computational grid in case of the AORCMs is often larger than the focus domain.

The ERA-Interim reanalysis^70^ constitutes the RCM forcing and the reference dataset, "REF", in the analysis. The REF data are retrieved from the ECMWF's Meteorological Archival and Retrieval System (MARS) on a global grid with a 0.75° resolution. From the "Synoptic Monthly Means" data stream ("mnth"), monthly means of the 12 h accumulated sums for the 00UTC and 12UTC forecast start times are retrieved as grib files, covering 1989 to 2008. Variables used are total precipitation [m of water equivalent] ("tp", parameter ID 228), defined as "the accumulated liquid and frozen water, including rain and snow, that falls to the Earth's surface. It is the sum of large-scale precipitation … and convective precipitation …" (https://apps.ecmwf.int/codes/grib/param-db?id=228), and evaporation [m of water equivalent] ("e", parameter ID 182), defined as "the accumulated amount of water that has evaporated from the Earth's surface, including a simplified representation of transpiration (from vegetation), into vapor in the air above" (https://apps.ecmwf.int/codes/grib/param-db/?id=182). As with the ALADIN RCM data, REF is remapped to the EUR-44 grid using cdo 1st order conservative remapping after a format conversion from GRIB to netCDF, including scaling and offsetting for unit conversion to mm year^−1^. The 0.75° resolution binary land-sea mask [0, 1] ("lsm", parameter ID 172) is resampled to the EUR-44 grid using a nearest neighbour resampling. Because there is nearly an exact match of the MED-44 and EUR-44 grids, the ERA-Interim reanalysis as prepared for the EUR-44 model domain is also used for MED-44, thereby reducing the Med-CORDEX model domain from 98 × 63 to 95 × 63 grid points. Given the larger deviations in model domain configuration and thereby differing spatial spinup zones with the Med-CORDEX MME, this simplification seems warranted.

### Observations

Precipitation observations are used as an independent dataset to illustrate the actual precipitation bias of the RCMs. The E-OBS dataset^72^ provides gridded daily precipitation data on a regular latitude–longitude grid at 0.1° and 0.25° resolution for Europe, based on the ECA&D (European Climate Assessment and Dataset) station data set, available through the Copernicus Climate Change Service. For this study, daily precipitation data [mm d^−1^] from the dataset version 21.0e of May 2020 is extracted using the cdo tools for the time span from 1989 to 2008, and temporally averaged to create long-term mean annual sums. A conservative remapping is applied to transfer the E-OBS data to the EUR-44 grid.

### Data processing and analysis methods

The RCM and REF simulation base data for the extraction of *P* and *E* timeseries over the complete model domain, ocean, and land areas are in [mm year^−1^] water equivalent data arrays. Before the timeseries extraction, all data are on the original equal area 106 × 103 EUR-44 grid (10,918 grid elements total), or 95 × 63 MED-44 grid (5985 grid elements total, truncated from 98 × 63 grid dimension). Only REF is regridded from the regular 0.75 degree grid to the EUR-44 grid, see above. Based on a land-sea mask threshold of ≥ 0.5 for land, time series of the spatial sums over the ocean and land areas, as well as the complete domain are derived per RCM and for the REF dataset applying to each model its own land-sea mask. The land-sea masks of the individual models differ slightly from each other due to the individual pre-processing of the RCM's static fields; for the EUR-44 grid the difference of the land grid points of the binary land-sea mask with the fewest land grid points to the one with the largest number of land grid points is 67 (0.6% of all grid points); the difference between the land grid points of the REF land-sea mask 5817 land grid points and the RCMs’ land-sea mask average 5858 land grid points is about 0.4%, due to the different spatial resolutions of the base data. The differences in the land-sea masks are considered small enough to neither justify a resampling of data to a common reference grid, taking into account a common land-sea mask, nor applying a weighing of spatial sums or means of precipitation and evapotranspiration based on the different number of land-sea mask grid points. For comparisons that involve the complete model domain or land or ocean areas exclusively, the spatial means of the annual sums are used (e.g., Figs. 3, 5a–c); in case ocean and land areas are compared, the means over the oceans and land areas are normalised by multiplying with the proportion of ocean and land points to the overall number of grid points, so that adding the weighted averages for the ocean and land areas yields the domain spatial average (Fig. 5d).

## Data Availability

The base data for the study (3D monthly and yearly data per RCM and REF on EUR-44 and MED-44 grid, extracted time series, land-sea masks, and base data for the plots, including the resampling weights for the REF data grid transformation) are available from the "Data Publication Server Forschungszentrum Jülich" under https://www.re3data.org/repository/r3d100012923.

## References

[1] Dickinson RE, Errico RM, Giorgi F, Bates GT (1989). A regional climate model for the Western United States. Clim. Change.

[2] Giorgi F, Bates GT (1989). The climatological skill of a regional model over complex terrain. Mon. Weather Rev..

[3] Giorgi F (1990). Simulation of regional climate using a limited area model nested in a general circulation model. J. Climate.

[4] Giorgi F (2019). Thirty years of regional climate modeling: where are we and where are we going next?. J. Geophys. Res. Atmos..

[5] Rockel B (2015). The regional downscaling approach: a brief history and recent advances. Curr. Clim. Change Rep..

[6] Rummukainen M (2016). Added value in regional climate modeling. Wiley Interdiscip. Rev. Clim. Change.

[7] Gutowski WJ (2020). The ongoing need for high-resolution regional climate models: process understanding and stakeholder information. Bull. Am. Meteorol. Soc..

[8] Mearns LO, Lettenmaier DP, McGinnis S (2015). Uses of results of regional climate model experiments for impacts and adaptation studies: the example of NARCCAP. Curr. Clim. Change Rep..

[9] Gutowski WJ (2016). WCRP COordinated Regional Downscaling EXperiment (CORDEX): a diagnostic MIP for CMIP6. Geosci. Model Dev..

[10] Bosshard T, Kotlarski S, Zappa M, Schär C (2014). Hydrological climate-impact projections for the Rhine River: GCM–RCM uncertainty and separate temperature and precipitation effects. J. Hydrometeorol..

[11] Kleinn J (2005). Hydrologic simulations in the Rhine basin driven by a regional climate model. J. Geophys. Res. Atmos..

[12] Naz BS (2016). Regional hydrologic response to climate change in the conterminous United States using high-resolution hydroclimate simulations. Glob. Planet. Change.

[13] Rasmussen R (2014). Climate change impacts on the water balance of the Colorado headwaters: high-resolution regional climate model simulations. J. Hydrometeorol..

[14] Rössler O (2019). Evaluating the added value of the new Swiss climate scenarios for hydrology: an example from the Thur catchment. Clim. Serv..

[15] Furusho-Percot C (2019). Pan-European groundwater to atmosphere terrestrial systems climatology from a physically consistent simulation. Sci. Data.

[16] Goderniaux P (2011). Modeling climate change impacts on groundwater resources using transient stochastic climatic scenarios. Water Resour. Res..

[17] Refsgaard J (2016). Climate change impacts on groundwater hydrology—where are the main uncertainties and can they be reduced?. Hydrol. Sci. J..

[18] Chilkoti V, Bolisetti T, Balachandar R (2017). Climate change impact assessment on hydropower generation using multimodel climate ensemble. Renew. Energy.

[19] Majone B, Bovolo CI, Bellin A, Blenkinsop S, Fowler HJ (2012). Modeling the impacts of future climate change on water resources for the Gállego River basin (Spain). Water Resour. Res..

[20] Wagner T (2016). Impacts of climate change on stream flow and hydro power generation in the Alpine region. Environ. Earth Sci..

[21] Hattermann FF, Huang S, Koch H (2015). Climate change impacts on hydrology and water resources. Meteorol. Z..

[22] Fowler HJ, Kilsby CG, Stunell J (2007). Modelling the impacts of projected future climate change on water resources in North-West England. Hydrol. Earth Syst. Sci..

[23] Christensen JH, Carter TR, Rummukainen M, Amanatidis G (2007). Evaluating the performance and utility of regional climate models: the PRUDENCE project. Clim. Change.

[24] Jacob D (2007). An inter-comparison of regional climate models for Europe: model performance in present-day climate. Clim. Change.

[25] van der Linden P, Mitchell JFB (2009). ENSEMBLES: Climate Change and Its Impacts: Summary of Research and Results from the ENSEMBLES Project.

[26] Mearns LO (2009). A regional climate change assessment program for North America. EOS Trans. AGU.

[27] Jacob D (2020). Regional climate downscaling over Europe: perspectives from the EURO-CORDEX community. Reg. Environ. Change.

[28] Kotlarski S (2014). Regional climate modeling on European scales: a joint standard evaluation of the EURO-CORDEX RCM ensemble. Geosci. Model Dev..

[29] Prein AF (2016). Precipitation in the EURO-CORDEX 0.11° and 0.44° simulations: high resolution, high benefits?. Clim. Dyn..

[30] Demory ME (2020). European daily precipitation according to EURO-CORDEX regional climate models (RCMs) and high-resolution global climate models (GCMs) from the High-Resolution Model Intercomparison Project (HighResMIP). Geosci. Model Dev..

[31] Hagemann S (2004). Evaluation of water and energy budgets in regional climate models applied over Europe. Clim. Dyn..

[32] Maraun D (2016). Bias correcting climate change simulations—a critical review. Curr. Clim. Change Rep..

[33] Teutschbein C, Seibert J (2012). Bias correction of regional climate model simulations for hydrological climate-change impact studies: review and evaluation of different methods. J. Hydrol..

[34] Ban N, Schmidli J, Schär C (2014). Evaluation of the convection-resolving regional climate modelling approach in decade-long simulations. J. Geophys. Res. Atmos..

[35] Knist S, Goergen K, Simmer C (2020). Evaluation and projected changes of precipitation statistics in convection-permitting WRF climate simulations over Central Europe. Clim. Dyn..

[36] Prein AF (2015). A review on regional convection-permitting climate modeling: demonstrations, prospects, and challenges. Rev. Geophys..

[37] Pieri AB, von Hardenberg J, Parodi A, Provenzale A (2015). Sensitivity of precipitation statistics to resolution, microphysics, and convective parameterization: a case study with the high-resolution WRF climate model over Europe. J. Hydrometeorol..

[38] Morrison H, Thompson G, Tatarskii V (2009). Impact of cloud microphysics on the development of trailing stratiform precipitation in a simulated squall line: comparison of one- and two moment Schemes. Mon. Weather Rev..

[39] Thompson G, Eidhammer T (2014). A study of aerosol impacts on clouds and precipitation development in a large winter cyclone. J. Atmos. Sci..

[40] Jaeger EB, Seneviratne SI (2011). Impact of soil moisture–atmosphere coupling on European climate extremes and trends in a regional climate model. Clim. Dyn..

[41] Schär C, Lüthi D, Beyerle U, Heise E (1999). The soil–precipitation feedback: a process study with a regional climate model. J. Climate.

[42] Rios-Entenza A, Soares PM, Trigo RM, Cardoso RM, Miguez-Macho G (2014). Moisture recycling in the Iberian Peninsula from a regional climate simulation: spatiotemporal analysis and impact on the precipitation regime. J. Geophys. Res. Atmos..

[43] Trenberth KE, Fasullo JT, Mackaro J (2011). Atmospheric moisture transports from ocean to land and global energy flows in reanalyses. J. Climate.

[44] Kjellström E, Ruosteenoja K (2007). Present-day and future precipitation in the Baltic Sea region as simulated in a suite of regional climate models. Clim. Change.

[45] Volosciuk C (2016). Rising Mediterranean Sea surface temperatures amplify extreme summer precipitation in central Europe. Sci. Rep..

[46] Weber T, Haensler A, Jacob D (2018). Sensitivity of the atmospheric water cycle to corrections of the sea surface temperature bias over southern Africa in a regional climate model. Clim. Dyn..

[47] Turuncoglu UU (2015). Identifying the sensitivity of precipitation of Anatolian peninsula to Mediterranean and Black Sea surface temperature. Clim. Dyn..

[48] Dimri AP (2012). Atmospheric water budget over the western Himalayas in a regional climate model. J. Earth Syst. Sci..

[49] Fersch B, Kunstmann H (2014). Atmospheric and terrestrial water budgets: sensitivity and performance of configurations and global driving data for long term continental scale WRF simulations. Clim. Dyn..

[50] Roberts J, Snelgrove K (2015). Atmospheric and terrestrial water balances of Labrador’s Churchill River basin, as simulated by the North American Regional Climate Change Assessment Program. Atmos. Ocean.

[51] Gao Y (2012). Moisture flux convergence in regional and global climate models: Implications for droughts in the southwestern United States under climate change. Geophys. Res. Lett..

[52] Keune J, Sulis M, Kollet S, Siebert S, Wada Y (2018). Human water use impacts on the strength of the continental sink for atmospheric water. Geophys. Res. Lett..

[53] Peixoto JP, Oort AH (1992). Physics of Climate.

[54] Davies HC, Turner RE (1977). Updating prediction models by dynamical relaxation: an examination of the technique. Q. J. R. Meteorol. Soc..

[55] Marbaix P, Gallée H, Brasseur O, van Ypersele J-P (2003). Lateral boundary conditions in regional climate models: a detailed study of the relaxation procedure. Mon. Weather Rev..

[56] Mesinger F, Veljovic K (2013). Limited area NWP and regional climate modeling: a test of the relaxation versus Eta lateral boundary conditions. Meteorol. Atmos. Phys..

[57] Beljaars ACM, Holtslag AAM (1991). Flux parameterization over land surfaces for atmospheric models. J. Appl. Meteorol..

[58] Sanchez-Gomez E (2011). Evaluation of Mediterranean Sea water and heat budgets simulated by an ensemble of high resolution regional climate models. Clim. Dyn..

[59] Di Luca A, Flaounas E, Drobinski P, Brossier CL (2014). The atmospheric component of the Mediterranean Sea water budget in a WRF multi-physics ensemble and observations. Clim. Dyn..

[60] Akhtar N, Brauch J, Ahrens B (2018). Climate modeling over the Mediterranean Sea: impact of resolution and ocean coupling. Clim. Dyn..

[61] Vautard R (2013). The simulation of European heat waves from an ensemble of regional climate models within the EURO-CORDEX project. Clim. Dyn..

[62] Ruti PM (2016). Med-CORDEX initiative for Mediterranean climate studies. Bull. Am. Meteorol. Soc..

[63] Batibeniz F (2020). Identification of major moisture sources across the Mediterranean Basin. Clim. Dyn..

[64] Williams CA (2012). Climate and vegetation controls on the surface water balance: synthesis of evapotranspiration measured across a global network of flux towers. Water Resour. Res..

[65] Trenberth KE, Smith L, Qian T, Dai A, Fasullo J (2007). Estimates of the global water budget and its annual cycle using observational and model data. J. Hydrometeorol..

[66] Kjellström E (2010). Daily and monthly temperature and precipitation statistics as performance indicators for regional climate models. Clim. Res..

[67] Vannière B (2019). Multi-model evaluation of the sensitivity of the global energy budget and hydrological cycle to resolution. Clim. Dyn..

[68] Laprise R (2008). Regional climate modelling. J. Comput. Phys..

[69] Cinquini L (2014). The Earth System Grid Federation: an open infrastructure for access to distributed geospatial data. Future Gener. Comp. Syst..

[70] Dee DP (2011). The ERA-Interim reanalysis: configuration and performance of the data assimilation system. Q. J. R. Meteorol. Soc..

[71] Schulzweida U (2019). Zenodo.

[72] Cornes R, van der Schrier G, van den Besselaar EJM, Jones PD (2018). An ensemble version of the E-OBS temperature and precipitation datasets. J. Geophys. Res. Atmos..

